# Methodological challenges to multivariate syndromic surveillance: a case study using Swiss animal health data

**DOI:** 10.1186/s12917-016-0914-2

**Published:** 2016-12-20

**Authors:** Flavie Vial, Wei Wei, Leonhard Held

**Affiliations:** 1 Veterinary Public Health Institute, Vetsuisse Faculty, University of Bern, Bern, Switzerland; 2 Epi-connect, Skogås, Sweden; 3 Department Biostatistics, Epidemiology, Biostatistics and Prevention Institute, University of Zurich, Zurich, Switzerland

**Keywords:** Applied statistics, Animal health, Laboratory, Syndromic surveillance, Multivariate, Temporal aberration detection, Outbreak detection, Outbreak prediction, Prospective surveillance

## Abstract

**Background:**

In an era of ubiquitous electronic collection of animal health data, multivariate surveillance systems (which concurrently monitor several data streams) should have a greater probability of detecting disease events than univariate systems. However, despite their limitations, univariate aberration detection algorithms are used in most active syndromic surveillance (SyS) systems because of their ease of application and interpretation. On the other hand, a stochastic modelling-based approach to multivariate surveillance offers more flexibility, allowing for the retention of historical outbreaks, for overdispersion and for non-stationarity. While such methods are not new, they are yet to be applied to animal health surveillance data. We applied an example of such stochastic model, Held and colleagues’ two-component model, to two multivariate animal health datasets from Switzerland.

**Results:**

In our first application, multivariate time series of the number of laboratories test requests were derived from Swiss animal diagnostic laboratories. We compare the performance of the two-component model to parallel monitoring using an improved Farrington algorithm and found both methods yield a satisfactorily low false alarm rate. However, the calibration test of the two-component model on the one-step ahead predictions proved satisfactory, making such an approach suitable for outbreak prediction. In our second application, the two-component model was applied to the multivariate time series of the number of cattle abortions and the number of test requests for bovine viral diarrhea (a disease that often results in abortions). We found that there is a two days lagged effect from the number of abortions to the number of test requests. We further compared the joint modelling and univariate modelling of the number of laboratory test requests time series. The joint modelling approach showed evidence of superiority in terms of forecasting abilities.

**Conclusions:**

Stochastic modelling approaches offer the potential to address more realistic surveillance scenarios through, for example, the inclusion of times series specific parameters, or of covariates known to have an impact on syndrome counts. Nevertheless, many methodological challenges to multivariate surveillance of animal SyS data still remain. Deciding on the amount of corroboration among data streams that is required to escalate into an alert is not a trivial task given the sparse data on the events under consideration (e.g. disease outbreaks).

## Background

Animal health surveillance provides scientific and factual evidence that is critically essential to inform decision making, and to motivate timely and appropriate animal health action [1]. An obvious specificity of animal health surveillance data is that they come from different species and different environments (e.g. wild animals, companion animals, and livestock). For the purpose of this paper, we will focus on the latter, i.e. through surveillance, we are interested in detecting health changes at all levels of the complex web that is a food production system, made up of sub-populations which are constantly moving around between farms to markets and slaughter.

Few stories illustrate the importance of early event detection for efficient disease control better than the 2001 foot-and-mouth epidemic in the UK. The 3 weeks delay in clinical detection of the virus by surveillance systems allowed the virus to spread across the country unnoticed. Ultimately, over 6.5 million livestock were slaughtered for disease control purposes, putting the estimated total cost of the epidemic at £8 billion [2]. Healthy animals contribute to public health by providing safe food and preventing contact between people and infectious animals carrying zoonotic pathogens. Healthy animals also contribute to strong rural agricultural economies. Being free from many animal diseases reduces the losses from disease (including lost animals and production), the cost for controlling diseases, and increases the quality of animal derived products, thereby making agriculture more competitive. Being free from animal diseases that prevent international free trade also opens market opportunities to farmers.

National veterinary services navigate “waters of budgetary restraint, strewn with the flotsam of competing issues”, in efforts to understand, control and eliminate complex disease situations [3]. Active surveillance methods, i.e., collecting data according to a defined plan relating to a particular condition, form the backbone of animal health surveillance systems and require significant investments in terms of resources and persons. Surveys to substantiate freedom from disease, routine serological surveillance for foreign animal diseases in sentinel farms or sampling of wildlife are all very costly and time-consuming. At the same time veterinary services are more and more confronted with limited resources. The development of cost-effective tools for animal disease and food safety surveillance is therefore of priority to decision-makers in the field of animal health [4]. Rapid advances in bioinformatics and data mining in the last decade have resulted in an increasing amount of data directly or indirectly related to animal health being collected and stored in very diverse databases. Some of these databases are curated by governmental organisations at national and regional levels; while others (e.g. production and reproduction data) are maintained by, and for the benefit of the livestock industry and affiliated businesses (e.g. veterinary practice). Most of these data are used for administrative or economic purpose. However, they could be used in the passive surveillance of animal health as part of an integrated syndromic surveillance (SyS) system. The goal of SyS is to monitor non-specific health indicators (termed “syndromes”) in a continuous real-time fashion in order to detect disease outbreaks earlier; and more rapidly characterise them than traditional notifiable disease methods [5]. Temporal aberrations in the occurrence of pre-defined syndromes are detected using algorithms that produce alerts when such syndromes occur more often than expected by chance. By definition, SyS does not target specific diseases as it is based on health data collected before a diagnostic has been made for individual cases. This is why alerts should be followed with epidemiological investigations that will identify the hazard present. Beyond outbreak detection, SyS, more generally, contribute to timely and accurate population health situation awareness.

Multivariate SyS systems (which concurrently monitor several health-related data streams) have greater sensitivity and are more reliable than univariate systems [6]. Not only does no single data source capture information from all the individuals involved in an outbreak; the information recorded is only partial and unspecific. For example, some diseases will cause a wide variety of clinical symptoms in different people or animals (e.g. diarrhea in some, fever in others) and/or will affect different strata of the population (e.g. different age or production groups). Since there is often different information contained in observations from different data sources, SyS systems should be multivariate by nature, i.e. simultaneously evaluating various combinations of multiple datasets. A SyS continuously screening multiple data streams may appeal to decision makers as consistent evidence may be used to suggest inferential accuracy.

Despite this demonstrated advantage [7], most operational SyS systems usually run multiple univariate aberration detection methods, each focused on detecting an unexpected increase in the times series of a particular syndrome (e.g. The Electronic Surveillance System for the Early Notification of Community-Based Epidemics ESSENCE II [8]). Univariate aberration detection algorithms have the advantage of ease of application and interpretation: they employ hypothesis testing to provide systematic alert protocols. However, univariate methods are less sensitive to changes in disease incidence and suffer from a higher rate of false alerts, causing users to ignore alerts when they occur all the time. Furthermore, most univariate aberration detection algorithms, such as statistical process control methods, cannot adequately deal with the specificities of surveillance time series such as the presence of strong seasonality or trends. These predictable effects must first be modelled and removed from the data, a step called pre-processing [9]. A historical baseline free of outbreaks is also necessary in order to be able to create models of expected behavior. This often constitutes a real challenge as most outbreaks may not be labelled, nor will their shape and magnitude be known [10]. On the other hand, a stochastic modelling-based approach to outbreak detection offers more flexibility, allowing for the retention of historical outbreaks, for overdispersion (low counts are typical), non-stationarity (e.g. decrease following an intervention) and the possibility to base the alarm system on the predictive distribution (i.e. performing outbreak prediction one time step ahead) [11]. While such methods are not new, they have only recently received attention in the field of public health surveillance [12] and are yet to be applied to animal health surveillance data.

The aim of this paper is to apply an example of such stochastic model, namely Held and colleagues’ two-component model [11], to two multivariate animal health datasets from Switzerland in order to 1) evaluate its performance under different properties- namely time alignment versus time mis-alignment of the events under surveillance; 2) and assess its calibrations under outbreak prediction surveillance scenarios. We start our Methods section by providing a succinct review of aberration detection methods (with a focus on multivariate methods), developed within the context of prospective SyS surveillance. We then explain the two-component model in more details before showing, in the Results section, its outbreak prediction performance compared to univariate models when applied to Swiss diagnostic laboratory data and cattle abortion data. In the Discussion, we share our experience of the methodological challenges encountered when attempting to apply multivariate methods to animal SyS data.

## Methods

### Prospective univariate surveillance

#### Outbreak detection

A comprehensive review of univariate statistical methods for prospective outbreak detection was compiled by [13]. In summary, the outbreak detection statistical process can be viewed as a two-step hierarchical model:

- In step 1, the data (e.g: counts, incidence rates, …) are modelled in order to obtain a prediction for each time point *t*, $Y_{t} \sim distr(\mu _{t}, {\sigma _{t}^{2}})$, where *μ*_*t*_ and ${\sigma _{t}^{2}}$ are the mean and variance. This step requires the training data to be free of historical disease outbreaks in order to determine ‘typical’ background behaviour against which the presence of abnormalities can be investigated [10].
- In step 2, the observed value at *t* is assessed against a prediction threshold, derived from the model in step 1, to define whether an alarm should be triggered.

Parametric regressions are commonly used in step 1. For example, Serfling’s method, a cyclic regression model, is capable of dealing with time trends and seasonal variations [14], and is the standard Center for Disease Control algorithm for flu detection. The method developed by [15], based on a quasi-Poisson regression, is routinely used by Health Protection Agency in UK. Semiparametric methods, such as the combined use of Gaussian kernel smoothers with generalised additive models as in [16], are also available. The threshold (*u*_*t*_) above which an alarm is triggered in step 2, can be defined in different ways: it can be defined as the upper prediction limit $u_{t} = \hat {\mu }_{t} + k \hat {\sigma }_{t}$ (where *k*>0 is a parameter to control the size of the aberration), or based on $\frac {2}{3}$-power transformation in the methods of [15, 17].

Statistical process control (SPC) charts also have a long history of application in public health surveillance. Cumulative sums (CUSUM), Exponentially Weighted Moving Averages (EWMA), and Shewhart control charts are routinely used for early disease detection on data from diagnostic laboratories in Sweden and parts of Canada [18].

Assuming that *Y*_*t*_ follows a normal distribution, $Y_{t} \sim \mathrm {N} (\mu _{t}, {\sigma _{t}^{2}})$, the one-sided (standardised) Gaussian CUSUM at *t* is defined iteratively by

1 $$ C_{t} = \max \left(0,C_{t-1} +\frac{y_{t}-\mu_{t}}{\sigma_{t}} -k \right),  $$

where *C*_0_=0, *k*>0 and the threshold *h* are the two constants that depend on the size of the aberration of interest. The parameter *k* is often chosen to be 1/2 [19] while *h* will depend on the desired false alarm rate. A CUSUM approach for count data [20–23] is implemented in R: surveillance [24] and R:vetsyn [25] packages.

#### Outbreak prediction

Outbreak prediction algorithms differ from their outbreak detection counterparts in two major ways. First, models are fitted using the whole historical data available, i.e. without removing data linked to past disease outbreaks. They have the feature to model epidemics. Second, the alarm system is based on the predictive distribution itself instead of comparing the observed value to a threshold. One example is the two-component model proposed by [11] for univariate analysis, later extended to multivariate modelling by [26]. The two-component model can be based on Poisson- or negative binomial distributed observations. Its first component is parameter-driven and links latent endemic seasonal patterns to disease incidence. Its second component is observation-driven and is based on the number of disease cases at previous time points.

Outbreak prediction has many advantages over outbreak detection. Since in step 1, the model does not require the removal of disease outbreaks from the data, it is based on all available historic information, unlike many other algorithms that ignore a large percentage of the data in order to avoid having to deal with seasonal effects. Furthermore, selection of a suitable model can be performed through the Akaike information criterion (AIC) or the Bayesian information criterion (BIC). The quality of the predictions can be assessed based on several diagnostic tools. Proper scoring rules have been applied in [27] to evaluate the predictions, which measure the predictive quality by assigning a numerical score, based on a stated predictive distribution and the later observed true value. The scoring rules are preferred by [28] to check the predictions from mixed models which include random effects for multivariate modelling. There are three commonly used proper scoring rules: the logarithmic score (LS) [29], the Dawid-Sebastiani score (DSS) [30] and the ranked probability score (RPS) [31, 32]. LS is the negative log-likelihood evaluated at the actual observation; DSS is the standardised difference between the actual observation and predictive mean value plus a penalty of the predictive variance; RPS is the sum of the Brier scores [33] for binary predictions at all possible thresholds. Calibration tests are developed in [34] to check the statistical consistency between observed values and their predictions. Such tests can be constructed based on the proper scoring rules and interpreted so that small *p*-values signify poorly calibrated predictions.

In step 2, outbreak prediction methods will raise a statistical alarm earlier than outbreak detection methods. An alarm is triggered based on the prediction instead of comparing the observed number with a predefined threshold. An alarm can be triggered at time *t* if the predictive probability that more than *u*_*t*_ cases will be reported is bigger than *θ*:

2 $$ \Pr(Y_{t} > u_{t}) > \theta.  $$

Here, the two threshold *u*_*t*_ and *θ* can be chosen to control the false alarm rate or be based on expert opinion. Alternatively, an alarm can be triggered based on a sequential Monte Carlo change point algorithm as in [35, 36]. This method can be applied to the epidemic component which is allowed to change over time [27] from one-step-ahead forecast distributions. Sequential particle filter algorithms have been applied in this setting in [37].

### Multivariate surveillance

#### Disease mapping and clustering

Point-pattern methods on spatio-temporal data can be used to assess whether disease cases are clustered, to identify areas of elevated disease risk and to generate hypotheses regarding disease etiology [38]. Numerous methods of spatial cluster detection have been published (see [39] for a review), including spatial point process summary methods [40], nearest neighbour methods [41], and local rate scanning methods [42]. Most of these methods can be implemented within freely available software: the *K* function and kernel intensity function within R [43], Cuzick and Edwards’ method [44] within ClusterSeer®; (BioMedware Inc. 2011), and Kulldorff’s scan statistic [45] within SaTScan [46].

A multivariate scan statistic was developed in software SaTScan to allow the detection of clusters in either a single or multiple datasets (without having to pre-specify which ones). Such multivariate approach can be useful to detect different sources related to the same pathogen or whether a common environmental hazard exists. Space-time scan statistics have further been extended using Bayesian methods as in the multivariate Bayesian scan statistics [47]; the Bayesian-network-based spatial scan statistic [48]; and the anomalous group detection methods [49].

Multivariate disease models may be considered to describe the space-time behaviour of diseases when the different datasets are correlated (influenced by common confounding factors). The multivariate conditional autoregressive (MCAR) model [50] and the shared component model [51] are the two main approaches to model disease risk correlations across both spatial units and diseases. However, spatial surveillance is only a special case of multivariate surveillance. Comparatively speaking, non-spatial data aggregation methods have received little attention.

#### Parallel monitoring of multivariate series

Parallel monitoring is often used on time series of a similar syndrome originating from multiple data sources, assuming that all the time series are independent and ignoring the correlation and interaction structure among them. Aberration detection algorithms are applied separately to each time series and an alert may be raised depending on how many statistics exceeds a limit and how the separate results are combined. The statistical challenge is to retain sensitivity while limiting the number of false alerts arising from multiple testing. Multiplicity adjustments are necessary to control the overall type I error, by applying a Bonferroni-type procedure [52] for example. A variant of parallel monitoring consists in combining the *p*-values to produce a single *p*-value by applying Edgington’s consensus method [53] for multiple experiments, or Fisher’s method of *p*-value aggregation [54]. Other authors propose rules to combine alarms [7]; the use of detection limits to generate an overall score representing the “severity” of any alarm [55]; or an approach using Bayes Belief Networks for combining univariate algorithm output [56]. Yet a different approach, [57] validated the use of multiple sources of routinely collected data in order to develop a weighting score system, the Continuous Cattle Health Monitor, which can be used to systematically (quarterly in this study) detect herds with poor cattle health, and direct surveillance efforts.

The main drawbacks of parallel monitoring are as follows:

- the different time series must be independent from one another, an assumption which is often violated with surveillance data. In our example, it would be fair to assume that the numbers of test requests received by the different diagnostic laboratories are not independent from one another.
- no extra knowledge about the health situation in the population is be gained when considering multiple sources of evidence in parallel (instead of jointly).
- the rate of false positives increases with the number of time series monitored.

The problem of multiple testing over units has been addressed by [58], as well as time by estimating the false discovery rate (FDR-proportion of all alarms detected that are false) for multiple units over a fixed surveillance period. As an alternative, a signalling procedure has been proposed by [59] to control the FDR. SPC critical thresholds depend on the data and thus change over time, “which can make the technique difficult to justify to a general audience” [60].

Even when spatial monitoring is not explicitly applied, the monitoring of multiple, parallel time-series can be used to deal with the availability of data over large spatial scales, with counts being grouped for specific regions. For example, [61] used hierarchical time series statistics, which account for the hierarchical spatial structure when grouping observations into different geographical scales. [62], on the other hand, monitored mortality rate in each of 1125 hexagons, into which France had been divided, and identified, weekly, which hexagons presented excessive mortality compared to the expectation of a Poisson regression model calibrated with historical data. They then applied special scan statistics to detect clusters of hexagons with high mortality.

#### Dimension reduction

An alternative to monitoring a large number of univariate time series is to try and reduce the number of random variables considered. Several approaches have been suggested to achieve a reduction of dimensionality. The sum or another linear combination of the variables, or principal components may be used instead of the original data variables [63, 64]. SPC methods are then applied to this single statistic [65]. The work presented in [66] would be a recent example of dimension reduction method used in animal SyS. In this example, assuming that the three sources of evidence were conditionally independent, the time series of nervous syndromes in horses, the time series of mortality in adult horses and the time series of the number of necropsied wild birds were added to provide evidence for or against the hypothesis of a West Nile outbreak.

Dimension reduction methods applied to surveillance suffer from several drawbacks: 1) by combining the information contained across all variables; we may lose some valuable health-related information contained in only one time series. For example, samples from an aborted cattle foetus sent to diagnostic laboratories may be tested for bovine viral diarrhea, infectious bovine rhinotracheitis, Q-fever or brucellosis as all four diseases can cause abortions. It is possible to reduce the dimension of our surveillance by combining the counts from all four time series and applying SPC methods. This aggregated time series would still allow us to detect early signs of an abnormal increase in the number of abortions which may signify a disease outbreak. However, the aggregated time series will not provide us with the information of which test request(s) increase, the knowledge of which would impact the ensuing epidemiological investigation and potential containment measures. 2) The variables monitored may be recorded using different units and it may not be possible to find a common scale. 3) Even when the variables are recorded on the same scale, they may exhibit different relationships to the underlying unobserved disease process. For example, daily counts of cattle births and cattle deaths should not be aggregated as most diseases would lead to a decrease of the former and an increase of the latter. 4) Finally, the covariance matrix of the input data streams will highly depend on a recent estimate [67]. More sophisticated methods of dimension reduction, termed “sufficient reduction”, will not reduce the information but still allow a joint solution to the full surveillance problem. However, the sufficient reduction solution cannot be applied to all multiple data streams, some further discussion can be found in [68].

#### Vector accumulation

The accumulated information from each time series is used by a transformation of the vector of component-wise alarm statistics into a scalar alarm statistic [65]. Multivariate SPC methods [69], such as MEWMA [70] (Lowry, Woodall, Champ, and Rigdon, 1992) and MCUSUM [19, 71] fall into this category. They are based on the assumption that the data or their residuals follow the normal distribution although a rank-based method or non-parametric scheme are discussed in [72, 73]. Applications in the field of veterinary public health and surveillance can be found in [74, 75].

#### Joint modelling methods

Joint modelling of the original data without dimension deduction or stepwise procedures is preferable when possible. Optimal methods can be constructed based on the full likelihood function of multiple series when known; although it can be complicated or require many assumptions. To counter this, a multivariate model has been developed by incorporating a branching process for count data in [76]. This model is capable of handling seasonal effects, time trends as well as spatio-temporal interactions (using auto regressive and neighbourhood effects). This model was further extended to analyse surveillance data of several pathogens [26], and an application to animal health surveillance can be found in [77, 78]. For a recent review and details on software see [79].

## Results

### Number of postcodes sending test requests to two laboratories

Laboratory test requests can be suitable for animal SyS [55] if laboratories collect and store the data in an automated and electronic way such as is done in Switzerland. Using data from the Swiss Federal Food Safety and Veterinary Office, we produced time series of the daily number of unique postcodes from which animal samples were sent to the largest two animal diagnostic laboratories (termed A and B) between 2008 and 2010. Only the data from weekdays were modelled (laboratories rarely process test requests at the weekend). The historic sample identification notation system used did not allow us to establish a direct link between samples and individual cattle. A veterinarian may send 5 samples to a laboratory all originating from the same animal but being tested for 5 different pathogens. Grouping the samples by postcode of origin allowed us to make such a link indirectly, although it is not possible to currently differentiate between samples coming from different individuals within the same herd or same municipality. The data were split into two parts: a training set (2008 and 2009) used for model learning and a validation set (2010) for outbreak detection or prediction.

**Fig. 1:**
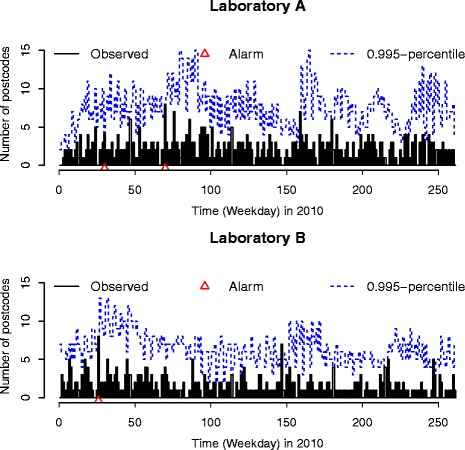
Outbreak detection. Statistical alarms (*red triangles*) raised by the improved Farrington algorithm applied to the time series of test requests (*black bars*) for laboratories A and B in 2010. Alarms are raised when the number of test requests on a given day exceeds the 0.995 percentile (*blue dashed line*)

The improved Farrington’s method [17] was applied to these two time series (Fig. 1), using the surveillance::farringtonFlexible function [24] in the R:surveillance package, to illustrate the application of parallel monitoring methods to the outbreak detection problem. The algorithm takes range values of the surveillance time series and for each time point uses a quasi-Poisson regression with overdispersion to compute a reference distribution for the current number of cases under an outbreak-free scenario - see [15, 17] for details. A quantile, say the 99% quantile, of this reference distribution then served as threshold to determine if the actual observation is unusually high: If the observation is above the bound, then an alarm is raised. In order to control the overall type I error, we applied a Bonferroni adjustment and defined the upper bound based on the 99.5*%* percentile corresponding to a 0.005 significance level for each time series, half of the significance level 0.01 used in [17]. Data from the same weekday (e.g. Monday, Tuesday,..., Friday) in the previous five weeks was included as historical data. An alarm was flagged (denoted by a red triangle on Fig. 1) on days for which the observed count was higher than the upper prediction threshold (blue line). Under these settings, two alarms were flagged for laboratory A and one alarm in laboratory B. Noting that one of the two alarms for laboratory A was close in time to the alarm raised for laboratory B, we decided to investigate possible interdependencies between the two time series using a joint modelling approach.

To that effect, we applied the two-component model to the laboratory data to predict outbreaks as discussed in Section “Outbreak prediction”. The full likelihood was assumed to follow a negative binomial distribution conditional on previous data, and the two time series were assumed to be conditionally independent. We denoted by *X*_A,*t*_ and *X*_B,*t*_ the number of cases in Laboratory A and B, respectively, in weekday *t*, conditional on the number of cases *x*_A,*t*−1_ and *x*_B,*t*−1_ one weekday earlier (with time index *t*−1). The joint model was defined as

3 $$\begin{array}{*{20}l} X_{\mathrm{A},t} | x_{\mathrm{A},t-1}, x_{\mathrm{B},t-1} &\sim \text{NBin} (\mu_{\mathrm{A},t}, \psi_{\mathrm{A}})\\ X_{\mathrm{B},t} | x_{\mathrm{A},t-1}, x_{\mathrm{B},t-1} &\sim \text{NBin} (\mu_{\mathrm{B},t}, \psi_{\mathrm{B}}), \end{array} $$

where *ψ*_*A*_ or *ψ*_*B*_ were the overdispersion parameters. The means were modelled as

4 $$\begin{array}{*{20}l} \mu_{\mathrm{A},t} &= \nu_{\mathrm{A},t} + \lambda_{\mathrm{A}} \, x_{\mathrm{A},t-1} + \phi \, x_{\mathrm{B},t-1}  \end{array} $$

5 $$\begin{array}{*{20}l} \mu_{\mathrm{B},t} &= \nu_{\mathrm{B},t} + \lambda_{\mathrm{B}} \, x_{\mathrm{B},t-1} + \phi \, x_{\mathrm{A},t-1}, \end{array} $$

where *ν*_A,*t*_ and *ν*_B,*t*_ were the endemic components, *λ*_A_ *x*_A,*t*−1_ and *λ*_B_ *x*_B,*t*−1_ were the epidemic components considered as autoregressive effects, and *ϕ* *x*_A,*t*−1_ and *ϕ* *x*_B,*t*−1_ referred to the neighbourhood component representing the interactive effects from the other laboratory. The endemic component *ν*_*j,t*_ was modelled by including the time trend, yearly fluctuation and a weekday effect [80]:

6 $$\begin{array}{*{20}l}{} \log \nu_{j,t} &= \alpha_{j,t} t + \sum_{s=1}^{S} (\delta_{s} \sin (\omega_{s} t) + \gamma_{s} \cos (\omega_{s} t)) \\ & \quad + \!\alpha_{j,\text{Monday}} + \!\alpha_{j,\text{Tuesday}} + \alpha_{j,\text{Wednesday}} +\! \alpha_{j,\text{Thursday}}  \\ & \quad + \alpha_{j,\text{Friday}}, \end{array} $$

where *j*=A or B and *ω*_*s*_=2*π* *s*/260, because there are 260 weekdays in one year. The data from the two laboratories shared the same seasonality, as modelled by a sine/cosine function. The neighbourhood effect parameter *ϕ* was assumed to be equal because we assumed the interactive effect was similar in both directions between Laboratory A and B. In contrast to outbreak detection, model selection based on Akaike information criterion (AIC) was possible, because it was based on the all historical data from 2008 to 2009. The model with *S*=2 was selected with the smallest AIC (for *S*=1,2,3, the corresponding AICs were 5151.9, 5148.8 and 5151.6).

**Fig. 2:**
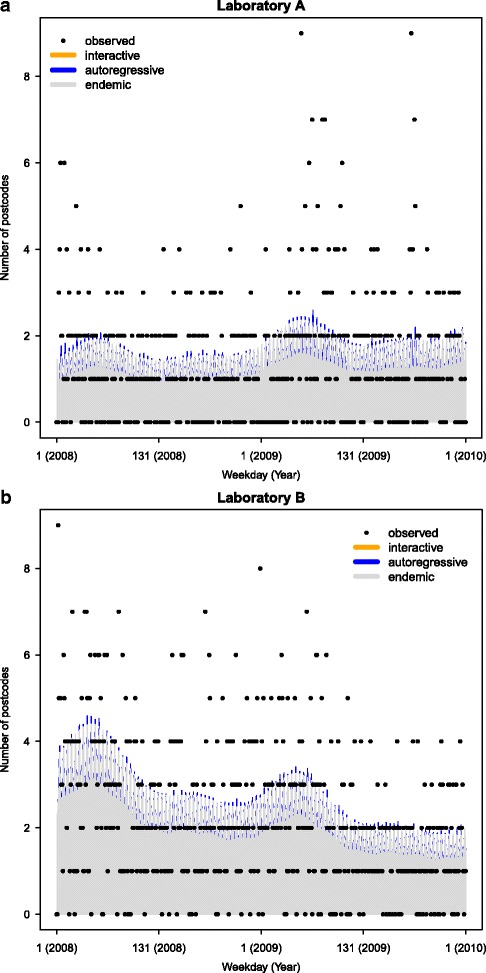
Components of joint model. Observed daily number of test requests (*black circles*) and fitted values from the joint two-components model applied to data from laboratories A (**a**) and B (**b**). The fitted values can be decomposed into three components: an endemic component (*grey*), an epidemic/auto-regressive component (*blue*) and a neighbourhood/interactive component (*orange*). In this example, the interactive component is weak (close to zero) and as such not clearly visible

The effect of the different components in such a model can be quantified to further understand disease dynamics. Parameter estimates from the model with *S*=2 are shown in Table 1. Parameters in the upper part of the table were fixed at the same value for both Laboratory A and B, while the laboratory specific parameters are shown in the lower part. The decomposition of the fitted values into the endemic (in grey color), the epidemic/autoregressive (in blue) and the neighbourhood/interactive (in orange) components (upper panel) are shown in Fig. 2 a & b, while the observed numbers are plotted as dots. Both time series exhibited a time trend: the number of unique locations requesting tests increased over time for laboratory A (*α*_*t*_=0.001) but decreased over time for laboratory B (*α*_*t*_=−0.001). No evidence for an interaction was found (*ϕ*=0). The parameters *ψ* for laboratory A and B were large compared to the predictive means from the models, indicating that there is no strong overdispersion in the data.

**Table 1:** Estimates with standard error (S.E.) of the parameters from the joint two-component model (*S*=2) in Eqs. (4) - (6) applied to the daily numbers of postcodes sending test requests to two diagnostic laboratories

Parameter	Laboratory A			Laboratory B	
	Estimate	S.E.		Estimate	S.E.
*ϕ*	0.000	0.000			
*δ*_1_	0.149	0.031			
*γ*_1_	0.063	0.031			
*δ*_2_	0.057	0.030			
*γ*_2_	−0.058	0.030			
Laboratory specific					
*λ*	0.042	0.035		0.030	0.036
*α*_*t*_	0.001	0.000		-0.001	0.000
*α*_Monday_	-0.106	0.107		1.293	0.075
*α*_Tuesday_	0.165	0.098		0.976	0.095
*α*_Wenesday_	0.364	0.095		0.833	0.093
*α*_Thursday_	-0.011	0.110		0.882	0.086
*α*_Friday_	-0.109	0.107		1.068	0.081
*ψ*	7.388	2.187		29.391	23.563

Parameters for Laboratory B in the upper part are empty because they are the same as those for Laboratory A: *ϕ* is the interactive parameter from the other laboratory; *δ*_1_ to *γ*_2_ are the parameters of sine and cosine function to adjust for the seasonal pattern. In the lower part of the table, parameters are different between Laboratory A and B: *λ* is the auto-regressive parameter; *α*_*t*_ is the time trend; *α*_Monday_ to *α*_Friday_ refer to weekday effect and *ψ* is the overdispersion parameter

A model-based approach to outbreak prediction would now be based on the one-step-ahead probabilistic forecasts. Good quality of the predictions is essential for the appropriateness on this approach. Proper scoring rules are commonly used to evaluate the quality of probabilistic forecasts [32]. A scoring rule imposes a penalty on the difference between the observation and the point prediction, the smaller the value is, the better the forecast is. Here the one-step-ahead predictions for the year 2010 have been evaluated using three commonly used proper scoring rules: LS, DSS and RPS. Generally it is very difficult to interpret the score value and the difference of the two scores. Hence we apply calibration tests based on these scoring rules to investigate if there is evidence for miscalibration [34, 81]. The *p*-values from the three calibration tests based on RPS, LS and DSS were large (0.60,0.93,0.59 for Laboratory A and 0.61,0.64,0.47 for Laboratory B). The *p*-values did not show any evidence of the predictions from this model being poorly calibrated, thereby validating our choice of a model-based outbreak prediction approach.

**Fig. 3:**
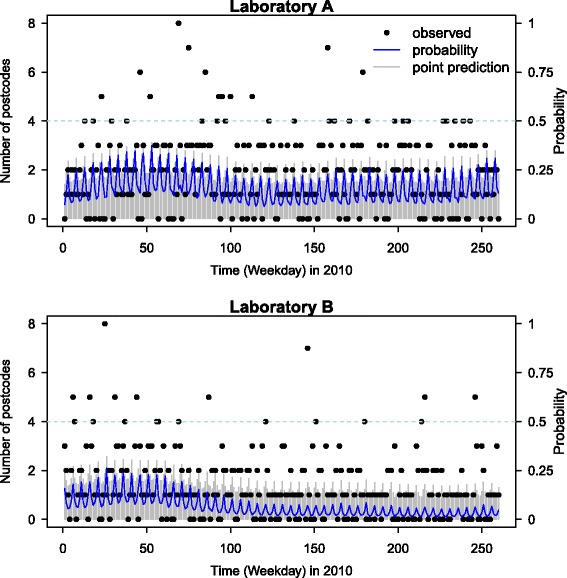
Outbreak prediction. One-step ahead predictions (*grey line*) and the probability that more than three locations send test requests to a laboratory on a given day (*blue line*) are shown. A statistical alarm would be raised should that probability be over 0.5 (none were raised for 2010)

Figure 3 illustrates the one-step-ahead predictions for the year 2010 as well as the alarms generated from the joint model. The grey bar shows the mean of the predictive distribution at each time point while the black dots are the observed numbers. The blue line is the probability of more than three locations sending test requests the following weekday. According to the alarm definition in Section “Outbreak prediction”, we set *u*_*t*_=3 and *θ*=0.5 in (2). In Fig. 3, the predictive numbers were plotted as grey bars and the observed number as black dots, the probability that Pr(*Y*_*t*_>3) was plotted as a blue line. If Pr(*Y*_*t*_>3)>0.5, an alarm is flagged. No alarms were raised in either time series. In practice, the threshold of probability applied in practice could be chosen based on expert opinion. Increasing it will further decrease the false alarm rate, while decreasing it will increase the model’s sensitivity.

### Joint modelling of time series with misalignment

An outbreak of disease in the population would produce signals in the time series at different times. In reality, the data time lag will often be different in different data sources. This is true for public health data but perhaps even more common in animal health surveillance data. A disease outbreak (e.g. in dairy cows) is best conceived as a chain of events through time: we may first observe an increase in the number of animals presenting fever, followed a few weeks later by an increase in the number of abortions reported, and even later an increase in the number of carcasses condemned at slaughter. The optimal surveillance method may depend on both the dependency between the monitored processes and the correlation between the time points when the changes occur [82]. For example, parallel surveillance works well when the changes occur far apart but a dimension reduction approach [83] is optimal when the changes occur very close in time to one another. For intermediate, but known, time-lags, an approach combining reduction by time and reduction by variable should be explored [65]. This “time alignment issue”, discussed in [6], poses a real problem as such delays are hard to quantify and will vary depending on the disease and/or the population. The first step would be to explore the lagged correlations among the time series. Lagged correlation between time series can be estimated in the absence of outbreaks by making some assumptions [56, 84]. But it may not be representative of true correlation in the presence of disease.

Let’s illustrate with an example from animal health surveillance. Because of processing and transporting delays, it is reasonable to expect that the number of samples (e.g. aborted material) sent to official diagnostic laboratories will increase later than the number of abortions recorded on farms. The research question of interest is to quantify how large such time lag is. We extracted the daily number of cattle abortions from the Swiss system for the identification and registration of cattle [85] and the daily number of test requests for bovine viral diarrhea (BVD- a disease that often results in abortions) sent to all official diagnostic laboratories between 2009 and 2011. The joint model was applied in order to determine the time misalignment between the two series denoted as *X*_Abor,*t*_ for the number of abortions and *X*_Test,*t*_ for the number of test requests. Similar to the method in Section“Number of postcodes sending test requests to two laboratories”, *X*_Abor,*t*_ and *X*_Test,*t*_ followed negative binomial distributions NBin(*μ*_Abor,*t*_,*ψ*_Abor_) and NBin(*μ*_Test,*t*_,*ψ*_Test_) respectively conditional on the previous data with

7 $$\begin{array}{*{20}l} \mu_{\text{Abor},t} &= \lambda_{\text{Abor}}\, X_{\text{Abor},t-1} + \phi_{\text{Abor}} \,X_{\text{Test}, t-r} + \nu_{\text{Abor},t},\\ \mu_{\text{Test},t} &= \lambda_{\text{Test}}\, X_{\text{Test},t-1} + \phi_{\text{Test}}\, X_{\text{Abor}, t-r} + \nu_{\text{Test},t}. \end{array} $$

Neighbourhood effects allowed the interactive effects to be different with different lags *r* in both directions : *ϕ*_Abor_ from the number of tests to the number of abortions and *ϕ*_Test_ from the number of abortions to the number of tests. In this method we can include the interactive effect with lags *r*=1,2,…,5 and the lags included are set as the same for the two series for the sake of simplicity. The data from 2009 and 2010 were retained as a learning dataset while the data from 2011 were used as a validation dataset.

**Fig. 4:**
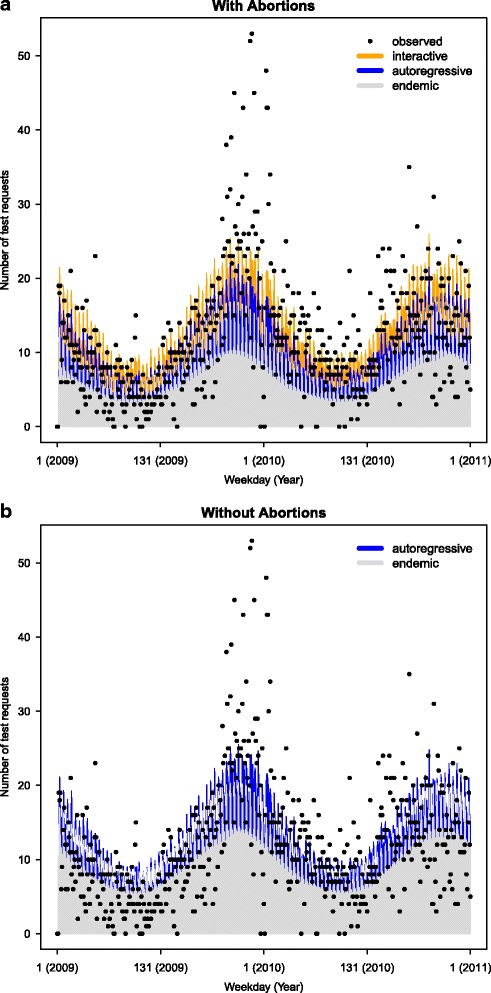
Joint modelling of time series with misalignment. Fitted values, and their decomposition into endemic (*grey*), epidemic (*blue*) and interactive (*orange*) components, from the joint model applied to the time series of the daily number of test requests for BVD with (**a**) and without (**b**) the lagged daily number of cattle abortions are shown

As in Section “Number of postcodes sending test requests to two laboratories”, the weekday effect and seasonality were included in the endemic component, for each time series *j*=Test or Abor,

8 $$\begin{array}{*{20}l}{}  \log \nu_{j,t} &= \sum_{s=1}^{S} (\delta_{j,s} \sin (\omega_{s} t) + \gamma_{j,s} \cos (\omega_{s} t)) \\ &\quad+ \alpha_{\text{Monday},j} + \!\alpha_{\text{Tuesday},j} + \!\alpha_{\text{Wednesday},j} + \!\alpha_{\text{Thursday},j}\\ &\quad+ \alpha_{\text{Friday},j}, \end{array} $$

and where *ω*_*s*_=2*π* *s*/260 (approximately 260 weekdays in one year). The best model retained the weekday effect and a seasonal effect with *S*=1 but no time trend (details are not shown here). Table 2 shows the estimated neighbourhood effects *ϕ* and their standard error. As expected, we did not find any evidence of an effect of the number of test requests on the number of abortions, since all estimates of *ϕ*_Abor_ were close to zero. However, there was some evidence for an effect of the number of abortions two days earlier (*r*=2) on the number of test requests with estimate *ϕ*_Test_=0.048 and 95*%* confidence interval (0.002,0.091). In addition, the model with *r*=2 fitted the data best according to AIC. We further compared this result with the univariate modelling of the number of test requests which included the same model as in (8) but without the neighbourhood effect. The fitting results from the two models are shown in Fig. 4 a & b. The proper scoring rules for the one-step-ahead predictions from the two models are shown in Table 3. The RPS and LS scores of the joint model were slightly smaller than those from the model without the number of abortions, indicating that the joint model is better. However, the differences in score values were not big and the mean DSS scores, rounded to two decimal places, were in fact the same. That is because the univariate model (with the overdispersion parameter *ψ*=4.99) accommodates larger forecast variances, making the average scores similar to the ones from the joint model (*ψ*=4.87). The overdispersion parameter in the joint model was larger, indicating that the predictions from the joint model are more precise than the ones from the univariate model. However, the *p*-values from calibration tests based on LS and DSS were fairly small for both models and suggested that the predictions are not well calibrated.

**Table 2:** The estimated neighbourhood effect *ϕ* of the joint two-components model (*S*=1) applied to the daily number of reported cattle abortions (Abor) and laboratory test requests (Test) for BVD. The different time lags *r* (in days) and the corresponding AIC values are presented

lag	*ϕ*_Test_(S.E.)	*ϕ*_Abor_(S.E.)	AIC
*r*=1	0.000(0.000)	0.000(0.000)	13002.9
*r*=2	0.048(0.022)	0.000(0.000)	12998.2
*r*=3	0.005(0.023)	0.030(0.056)	13000.3
*r*=4	0.017(0.021)	0.013(0.056)	13000.4
*r*=5	0.009(0.023)	0.000(0.000)	13000.9

**Table 3:** Average proper scores for the one-step-ahead predictions for the number of laboratory test requests from 1) the joint model accounting for the lagged number of reported abortions, and 2) the univariate model not accounting for the number of reported abortions

	With abortion		Without abortion
	Mean (*p*-value)		Mean (*p*-value)
RPS	3.71 (0.088)		3.74 (0.09)
LS	3.27 (0.002)		3.28 (0.003)
DSS	4.93 (0.002)		4.93 (0.003)

## Discussion

Defining the population under surveillance in animal SyS systems is often more complex than for public health surveillance. Such systems have to accommodate diverse species, production types within species (e.g. dairy versus meat-producing cattle), different entities of interest (e.g. individual or herd) and different professionals interacting with the population. In animal health, most early-warning systems are based on continuous collection and analysis of clinical and treatment data from various sources such as farmers themselves, veterinary practitioners, animal health services, specialised clinics, university hospitals, pharmaceutical companies and breeding organisations. For example, Sikava is the Finnish electronic pig register used for national pig health monitoring. Finnish pig farmers submit the numbers of animals and mortality rates, as well as drug use information, to Sikava’s medical records. Breeding farms have additional requirements for disease testings [86]. In Switzerland, no such centralised clinical and treatment recording system exist, i.e. such a system would need to be developed, at great costs before it could be used in SyS. As budget restrictions continuously force national veterinary services to seek reductions in the unit cost of assessing animals and animal clusters [3], alternative data sources are being explored for use in SyS. In Switzerland, these include the national systems for the identification and registration of cattle [85] and meat inspection data from slaughterhouses [87]. Animal SyS in Switzerland faces similar statistical issues to health care surveillance systems. A common theme is the variety of types of data: there may be standardised (mortality or incidence) ratios, proportions, counts of adverse events, categorical data and even qualitative ‘intelligence’ that may need to be aggregated up a hierarchy. Yet, as in healthcare surveillance, we experience “a demand for methods that are straightforward to implement, can be explained to multiple stakeholders and are robust to potentially mediocre quality data” [60]. Many research questions still present themselves. Here we consider four commonly encountered multivariate data scenarios that we believe are of importance but to which the multivariate outbreak detection or prediction methods presented above may not always be applicable.

### Different temporal granularity scenario

The time series have the same data type (e.g. counts) but different temporal resolution (e.g. daily, weekly, monthly). As animal SyS is based on a variety of indirect health indicators collected by a variety of stakeholders for purposes, often, other than surveillance, the different indicators to integrate in a multivariate SyS system may exhibit different temporal and spatial resolution. For example, in Switzerland, data on cattle births are recorded on a daily basis, while post-mortem meat inspection data are only available on a monthly basis. Aggregating all data sources to the largest resolution present (e.g. monthly) would make possible the use of the various multivariate methods described above but would reduce the timeliness of the system. Time series clustering methods may be applicable on series which exhibit a small difference in temporal resolution. They take into account time series trends during periods of interest and then performs a clustering analysis in order to highlight relationship among the different data and their trends. A novel approach has been developed by [88] for multivariate time series clustering using dynamic time series segmentation and Self Organizing Maps techniques, and applied to geophysical data (differing in their sampling rate) recorded from monitoring networks at Mt. Etna.

### Different spatial granularity scenario

The time series have the same data type (e.g. all counts) but different spatial resolution (e.g. farm-level, postcode level, canton). There is a large spectrum of scale and units at which animal SyS data are recorded. An individual animal (e.g. a cow or a horse) may be the unit of surveillance. The animal unit is a window unto the collective health status of its herd or flock. In some data sources, only a subset of animals may have been sampled (e.g. animals sent to slaughter) or the data from several animals may be pooled together (e.g. bulk milk testing). Surveillance data may also originate from an enterprise (e.g. a market through which animals from different herds pass) or only be available at the regional or national level. One option is to aggregate all spatial data to a common denominator (e.g. municipality) and to look for space-time clusters using a multivariate spatial scan statistic method as in [89].

### Different data types scenario

The time series have the same time resolution (e.g. all daily) but different data types (e.g. counts, rate, continuous). In the easiest surveillance scenarios, it may be possible to transform the count data streams into proportions or rates and apply the various multivariate methods described in Section “Methods”. Data standardization and aggregation based on Z-scores is also discussed in [60]. Another alternative would consist in performing univariate modelling of all the data streams and setting rules of thumbs regarding the interpretation of the statistical alarms (akin to the alarm scoring system proposed in [90]). However, complex modelling approaches based on Bayesian methodologies, such as the ones developed by [91], which are much more apt to discover the interplay among multiple and disparate syndromic data sources, will play a big part in the future of AHSyS in which ever more integrative information systems are put in place for disease surveillance across domains (One Health).

### Reporting delay and nowcasting

Health system users and decision makers depend on timeliness to take appropriate action based on the urgency and the type of responses required by the situation. However, such system is inherently “contaminated” by the reporting delay between the occurrence of the event, for example, time of symptom onset or visit by the veterinarian, and the time the report becomes available in the surveillance database. Ideally, surveillance systems should be able to deal with the occurred-but-not-reported-events problem [92]. Delay-adjusting tracking procedures, also called nowcasts, are developing in the public health setting [93]. Nowcasting methods are not limited to univariate surveillance scenarios. The Bayesian hierarchical models commonly used for forecasting [94] offer a very suitable framework for multivariate surveillance.

## Conclusions

The answer to the question “which multivariate surveillance method is the best?” is the same as the answer to the question “which univariate surveillance method is the best?”: different methods are suitable for different problems. The choice of which univariate method(s) to apply will be based on the characteristics of the time series, the length of historic data available and the type of outbreak the SyS system must detect (Section “Outbreak detection”). The choice of which multivariate method(s) to apply will be based, additionally, on whether all data are of the same type and granularity as well as on our knowledge of the dependency between the monitored processes and the correlation between the time points when the changes occur (Section “Multivariate surveillance”). Some diseases may lead to near-simultaneous increases in two or more syndrome monitored, in which case a reduction of dimensionality to a univariate surveillance problem is appropriate. On the other hand, if the syndromes monitored are expected to “respond” independently to the introduction of a pathogen in a population, parallel monitoring and joint modelling may yield more satisfactory results. We favour the latter. The model assumes that syndrome counts can be viewed as the sum of an endemic and an epidemic component. The proportion of the epidemic component *λ*_*t*_ is allowed to vary over time according to a Bayesian multiple change point model, allowing the user to model time with occasional outbreaks or other non-stationary features (e.g. decreased counts as a result of quarantine or other interventions) [11]. The stochastic modelling approach also offers more interesting possibilities of being extended to address realistic surveillance scenarios. For example, the inclusion of times series specific parameters, such as overdispersion *ψ* or seasonality when applied to multivariate time series [26]. It becomes also possible to relate the endemic incidence *μ* or the epidemic parameter *λ* to “ecological” covariates [76, 95] known to have an impact on syndrome counts (e.g. in [87] slaughterhouse size was significantly linked to carcass condemnation rates). A spatial extension of the model would relate the endemic incidence *ν*_*t*_ or the epidemic parameter *λ* to area-level covariates (e.g. in [87] carcass condemnation rates varied between geographical regions), or would be well suited to capture space-time dependence caused by the spatial spread of a disease over time [76]. Setting up operational multivariate SyS systems whether based on traditional outbreak detection algorithms or on more complex models, requires decision making about: (1) which combinations of data sources to test, (2) how to achieve sensitivity over many data streams while retaining manageable false alert rate frequency (see Section “Parallel monitoring of multivariate series”), and (3) how much corroboration among data streams is required to achieve a threshold for escalating the information into an alert that should require investigation [6]. The first point is related to the more general issue of the epidemiological evaluation of data sources used for health surveillance (see [87] on how to evaluate data sources for SyS). One important consideration is how well the data collected match the required surveillance coverage in terms of populations, production types and geographic regions. Another consideration is timeliness. This is particularly important for SyS systems whose objectives is the early-detection of unexpected health events. Not only should data be collected in near-real time on the unit of interest (e.g. farm, laboratory), they should also be reported to a central database in real-time for data aggregation and analysis or they will have little value for early disease detection. Evaluation for animal health surveillance systems and data exist [96, 97] and should be used to quantify the usefulness of health and production recodring systems at the national level (or whichever level the SyS system is operated at). It is important to recognise that timeliness should not be treated as the be all and end all of SyS. A fully electronic system built around a centralised database is still of limited value if the system is not reliable (functioning without fail) or accurate (complete and correct) [98] (see point 2 above). Finally, point 3 is not trivial given that the understanding of the relationships between different data streams can be poor when data are sparse and the events under consideration (such as disease outbreaks) occur infrequently.

The key difficulty in dealing with a multivariate system in which time misalignment occurs (see Section “Joint modelling of time series with misalignment”) resides in measuring the relationship between data sources when an outbreak occurs. For this, we need datasets with clearly identified and delimited (in time) health events. While such data may be more easily available or derived for large well-defined seasonal outbreaks, the use of a small number of variables to define the occurrence of an outbreak may constitute a limited indicator and result in smaller outbreaks being missed [99]. Alternatively, expert judgement may be used retrospectively to determine the occurrence of events of public health importance, often using resource-intensive epidemiological investigation techniques.

Going back to the applications of the joint model to data on laboratory test requests in Section “Number of postcodes sending test requests to two laboratories”, we note that the Farrington method has flagged three alarms in the two time series considered. In general, several definitions of what constitutes a statistical alarm are possible for multivariate outbreak detection. We could combine model/algorithm output from each series (each with its own threshold) in a manner akin to [7]. The combination process could follow a majority rule (an overall alarm is flagged if more than half of output signaled an alarm at time *t*), or a *M*+*N* rule (it is flagged only if all output in the subset *M* signaled an alarm, and at least n of the remaining output signal an alarm). Alternatively, an overall alarm may be triggered if the joint probability of an outbreak across all series is higher than a fixed threshold. Being able to identify why an alarm was raised by a multivariate surveillance system is essential to guide the subsequent epidemiological investigation. The first step in investigating an alarm is confirmation of the signal through the examination of individual cases that triggered the alarm to obtain geographic (and potentially demographic) data [100]. This step may be complicated for multivariate systems in which data streams were aggregated either spatially or temporally because of differences in their resolution. Then if the signal is determined to not be a consequence of duplication of individual case data or data entry error, the specificity of the signal needs to be increased by requesting additional testing to rule specific diseases in or out or by querying specialists about specific conditions relating to the excess cases for example. This second step highlights the importance of, and difficulty in, extracting the right sort of knowledge from multivariate health data to focus the health services resources after a statistical alarm is raised. One advantage of multivariate parallel surveillance is that the interpretation of alarms is clear. Other methods, like Hotelling *T* ^2^ control chart, are not capable of distinguishing a change in the mean vector from a change in the covariance structure for example [69].

We conclude that many methodological challenges to multivariate animal health SyS still remain. Some of these challenges may require a change in the legislation to be surmounted. For example, the ubiquitous lack of commonly adopted data standards, make difficult data integration across heterogeneous datasets and/or geographical regions and may only be overcome through an official act defining such standards. Other challenges may only be overcome after the SyS system has been operational for some time and lessons are learned by trial and error. For example, deciding on the amount of corroboration among data streams that is required to escalate a statistical alert into an epidemiological alert is a difficult decision to take a priori given the sparse data on the events under consideration (e.g., disease outbreaks). While some of the desired improvements in multivariate surveillance data collection may take some time to implement, animal health SyS experts are encouraged to already look into stochastic modelling-based approaches to outbreak detection which address more realistic surveillance scenarios than traditional methods such as SPC and offer more flexibility. Methods such as the two-component model allow for the retention of historical outbreaks; for overdispersion; for non-stationarity; for the inclusion of times series specific parameters, or of covariates known to have an impact on syndrome counts; and for the possibility to base the alarm system on the one-step-ahead predictive distribution rather than comparing the observed number with a predefined threshold, potentially expediting the detection of events of veterinary public health interest and the implementation of containment measures.

## Abbreviations

AIC: Akaike information criterion
BIC: Bayesian information criterion
BVD: Bovine viral diarrhea
CUSUM: Cumulative sums
DSS: Dawid-Sebastiani score
EWMEA: Exponentially weighted moving averages
FDR: False discovery rate
LS: Logarithmic score
MCAR: Multivariate conditional autoregressive
MCUSUM: Multivariate cumulative sums
MEWMEA: Multivariate exponentially weighted moving averages
MSPC: Multivariate statistical process control
RPS: Rank probability score
SyS: Syndromic surveillance
SPC: Statistical process control
